# Is Ozone a Valid Adjuvant Therapy for Periodontitis and Peri-Implantitis? A Systematic Review

**DOI:** 10.3390/jpm13040646

**Published:** 2023-04-08

**Authors:** Francesco D′Ambrosio, Mario Caggiano, Alfonso Acerra, Massimo Pisano, Francesco Giordano

**Affiliations:** Department of Medicine, Surgery and Dentistry, University of Salerno, 84100 Salerno, Italy; macaggiano@unisa.it (M.C.); pisano.studio@virgilio.it (M.P.); frgiordano@unisa.it (F.G.)

**Keywords:** ozone therapy, periodontal diseases, periimplantitis, periodontitis, ozone oil, ozone water, ozone gaseous, ozone

## Abstract

Introduction: Ozone is a naturally occurring unstable compound with three oxygen atoms that generally transforms into an oxygen molecule, releasing one oxygen atom. This feature has been exploited in dentistry for numerous applications, including for periodontal diseases and peri-implantitis. Methods: This review was performed in relation to the PRISMA flow chart and was annotated in the PROSPERO register. PICO questions were used as research questions. The risk of bias in the non-randomized clinical trials was appraised using the ROBINS-I tool. Results: An electronic search found a total of 1073 records, in particular, 842 from MEDLINE/PubMed, 13 from Bio Med Central, 160 from Scopus, 1 from the Cochrane library databases, and 57 from the PROSPERO register. A total of 17 studies were included in the present systematic review. Information regarding the characteristics of the periodontal clinical and radiographic parameters for gaseous ozone, ozonate water, ozonate oil, and ozone gel, including clinical attachment loss (CAL) probing depth (PPD), bleeding on probing (BoP), plaque index (PI), gingival index (GI), and marginal bone levels (MBL), were obtained. Conclusions: The studies included in this systematic review show different results regarding the ozone in periodontal treatment in association with or without SRP.

## 1. Introduction

Ozone is a natural compound and an allotropic form of oxygen as it is composed of three oxygen atoms. It is also called triatomic oxygen or trioxygen [1].

In nature, it is generated when ultraviolet rays strike oxygen atoms, causing them to temporarily recombine into groups of three. Ozone is found in the form of gas in the stratosphere where it performs the important function of absorbing harmful ultraviolet radiation from the sun from water [1]. Therefore, ozone in the stratosphere plays an important role, protecting life on the earth’s surface [2].

Ozone is an unstable gas that rapidly transforms into oxygen molecules, thus releasing an oxygen atom. This feature has been used to eliminate bacteria, fungi, to inactivate viruses and control bleeding and has therefore been exploited in medicine [3].

Ozone was already used at the end of the 1800s in the USA and then later also among German soldiers during the First World War for the treatment of post-traumatic gas gangrene, infected wounds, mustard gas burns and fistulas [4,5].

Today, ozone therapy is used in medicine and in dentistry where it is used for different applications and through various media such as gas, water, oil and gel. It is not used intravenously as an air embolism may form [6,7].

However, ozone therapy also has contraindications, which have been described by Nogales et al., such as pregnancy, ozone allergy, acute alcohol intoxication, severe anemia, autoimmune disease, active bleeding, hyperthyroidism, myasthenia gravis and myocardial infarction [8].

Due to its properties, it is used in medicine and dentistry both in the form of gas, dissolved in water, in the form of oil and recently also in gel, and it seems to perform a disinfectant action in the management of dental caries, gingivitis, periodontitis and in the disinfection of root canals. It is used to control bleeding and to clean wounds in bone and soft tissue in the management of osteonecrosis of the jaw and pain management [9,10]; it appears to be able to increase local oxygen supply to wound areas and thus improves and accelerates wound healing; and it has been proposed for rapid resolution of clinical symptoms in oral lichen planus, temporomandibular disorders and for sensitivity control and tooth whitening [3,7].

The efficacy of ozone has also recently been evaluated in adjuvant therapy for COVID-19, producing controversial results [11,12].

Periodontitis is a bacterial disease characterized by chronic inflammation, which progressively affects and destroys the supporting tissues of the teeth and, ultimately, leads to the loss of bones and teeth [13,14]. Similarly, peri-implantitis is a bacterial disease of the soft tissues surrounding an endo-osseous implant, and the inflammation that occurs as a response of the body to limit and fight the action of bacteria leads to progressive peri-implant bone loss up to implant failure [13,15,16,17].

Periodontitis and periimplantitis have a bacterial etiology, so it is interesting to understand the antibacterial efficacy of ozone, used in different formulations, alone or in combination with scaling and root planing (SRP) [18,19,20].

The gold standard for periodontal therapy is scaling and root planing (SRP), and many authors have proposed different adjuvant agents in combination with SRP, including antibiotics, probiotics, laser therapy and ozone [20,21,22,23,24].

The purpose of this review is to analyze the efficacy of ozone in periodontal disease and peri-implantitis and the feedback on clinical periodontal and peri-implant parameters.

## 2. Materials and Methods

### 2.1. Study Protocol

This review was performed according to the PRISMA (Preferred Reporting Items for Systematic Reviews and Meta-analyses) flow chart [25] and was annotated in the PROSPERO (International Prospective Register of Systematic Reviews) systematic review register (code ID: CRD42023397763), as recommended by Booth et al. [26].

PICO (Population, Intervention, Comparison, and Outcome) questions were used for the research questions [27].

The clinical question in the “PICO” format was: Is there a significant difference in clinical, radiographic and inflammatory parameters of periodontal and peri-implant tissues treated by ozone gaseous, oil or water with or without other interventions compared with placebo or other interventions?

P (Population): subjects with periodontal or peri-implant diseases.
I (Intervention): gaseous, oil or water ozone treatment alone or with other interventions.
C (Comparison): subjects treated with placebo or periodontal or peri-implant treatment without ozone.
O (Outcome): Clinical, radiographic and inflammatory periodontal and peri-implant tissue parameters.

### 2.2. Search Strategy

Studies published in the English language concerning ozone treatment using water and oil in periodontitis and in peri-implantitis were electronically searched without date restrictions until 1 February 2023 across major scientific online databases, including PROSPERO register, BioMed central databases, Scopus, MEDLINE/PubMed and the Cochrane Library databases by two authors (A.A and F.D.A.) using the following keywords combined with Boolean operators, as show below:

(periodontal disease OR periodontitis OR peri-implantitis OR dental OR peri-implant disease OR mucositis OR oral lesions) AND (ozone water OR ozone treatment OR ozone oil OR ozone OR gaseous ozone OR ozonide).

The following filters were applied:

- English language on the Scopus library and on the MEDLINE/PubMed database;
- Reviews, systematic reviews and metanalyses were excluded from the MEDLINE/PubMed database.

No filters were applied to the BioMed Central database, to the PROSPERO register or to the Cochrane library.

### 2.3. Study Selection and Eligibility Criteria

The inclusion and exclusion criteria that were established before the start of the search and adhered to when selecting studies are shown below:

Inclusion Criteria:

- Studies published in English;
- Studies with clinical and/or radiographic parameters;
- Adult studies.

Exclusion Criteria:

- Studies without clinical or radiographic parameters;
- Studies not published in English;
- Reviews or systematic reviews;
- Pediatric studies.

Titles and abstracts obtained from the search as described were screened independently by two reviewers (F.D.A. and A.A.).

The Zotero (Version: 5.0.96.3.)reference manager tool was used to eliminate duplicates, and the obtained titles were analyzed by two Authors (A.A and F.D.A) who independently read the abstracts of relevant studies.

From the abstracts that were judged to be the most relevant, the complete texts were obtained and independently screened by the authors. A third reviewer (F.G.) was consulted in cases involving doubts or disagreements.

Two authors also screened the bibliographies of the included reviews for any titles relevant to this umbrella review.

No restrictions were applied regarding the number or kind of studies included in this systematic review.

All studies analyzing treatments with gaseous, aqueous and oil ozone with periodontal parameters were considered in this review.

### 2.4. Data Extraction and Collection

Data were extracted by two reviewers (M.C. and A.A.) independently, and a third author was involved in case of disagreement (F.D.A.).

From each study included in this systematic review, the following data criteria were recorded:

∘ First author, year of article, reference and study design;
∘ Population, sample size and mean age of the study population of each study;
∘ Type and regimen of gas, water or oil application used in periodontal treatment in each study;
∘ Time points;
∘ Clinical outcomes recorded, including clinical attachment loss (CAL), probing depth (PD), bleeding on probing (BOP), plaque index (PI) and gingival index (GI);
∘ Radiographic outcomes recorded, including marginal bone levels (MBL) and cytokine profiles;
∘ Conclusions.

### 2.5. Data Synthesis

The characteristics are presented in tabular form and summarized through a narrative synthesis.

Data were synthesized using Microsoft Excel software 2019 (Microsoft Corporation, Redmond, WA, USA).

In particular, with this review, we wanted to accomplish the following tasks:

- Characterize the type and regimen of treatment performed with water or oil with ozone and make possible comparisons;
- Compare the clinical radiographic results and cytokine profiles and bacteria amounts after ozone treatment (gaseous, water or oil) alone or with other treatments vs. placebo or other treatments.

### 2.6. Quality Assessment

The risk of bias in the non-randomized clinical trials was assessed using the ROBINS-I (“Risk Of Bias In Non-randomized Studies-of Interventions”) tool [28].

In this tool, biases are classified as biases due to confounding, biases due to selection of participants, biases due to classification of interventions, biases due to deviations from intended interventions, biases due to missing data, biases in measurement of outcomes or biases due to selection of the reported result [28].

Low risk of bias: the study is judged to be at low risk of bias for all domains;

Moderate risk of bias: the study is judged to be at low or moderate risk of bias for all domains;

Serious risk of bias: the study is judged to be at serious risk of bias in at least one domain, but not at critical risk of bias in any domain;

Critical risk of bias: the study is judged to be at critical risk of bias in at least one domain.

The response options regarding bias are: Yes(Y), Probably yes(PY), Probably no(PN), No(N) and No information(NI). “Y” indicates a low risk of bias, PY indicates a moderate risk of bias; PN indicates a serious risk of bias, N indicates a critical risk of bias and NI indicates that there is no information related to bias.

## 3. Results

### 3.1. Study Selection

An electronic search identified a total of 1073 records. In particular, 13 records were found using BioMed Central, 160 records were found using Scopus, 842 records were found using MEDLINE/PubMed, 1 record was found using the Cochrane library databases and 57 records were found using the PROSPERO register.

In total, 96 duplicates were eliminated, and 977 title abstracts were screened.

Of these 977 title abstracts, only 29 abstracts were useful for the present systematic review, and of these 29 records, their full texts were obtained and screened, and 12 articles were excluded, as shown in Table 1.

The table above includes author names, years of publication of their studies and motivation for exclusion of their studies from the present review.

**Figure 1:**
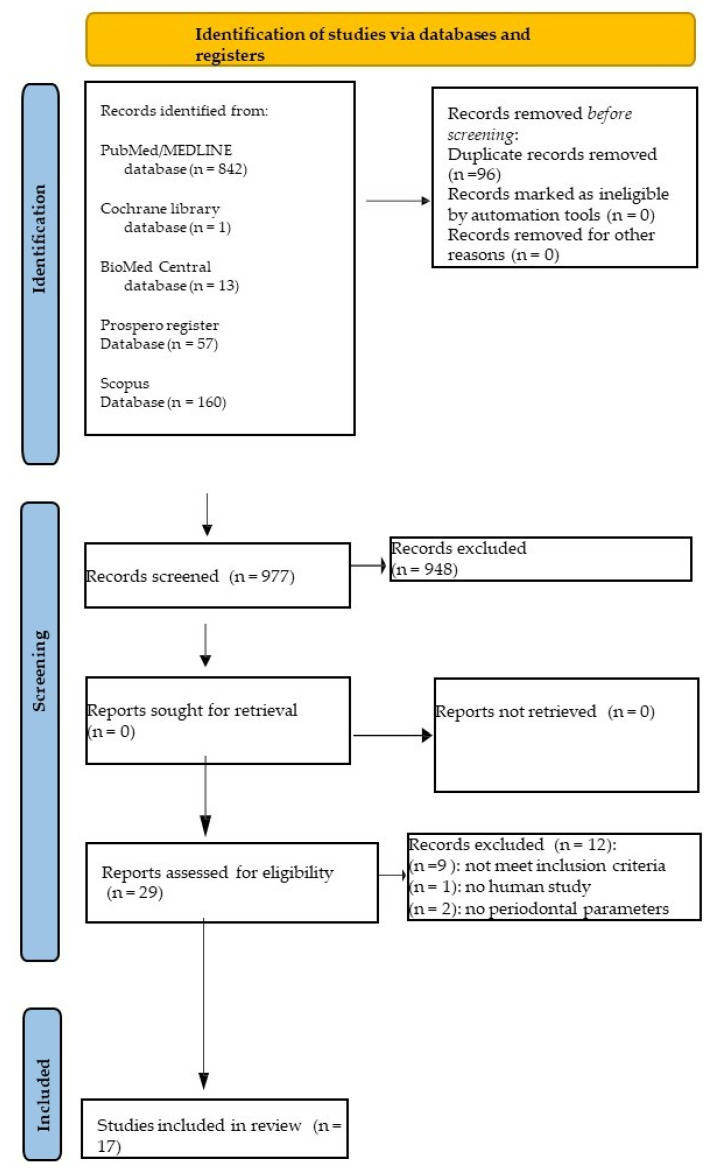
PRISMA flowchart of the screening process.

A total of 17 studies were included in the present systematic review (Figure 1).

### 3.2. Study Characteristics

The main features of the included studies are summarized in Table 2.

Of note, all included studies were published between 2010 and 2022 and were RCTs.

### 3.3. Data Extraction and Synthesis

Detailed findings related to periodontal clinical, radiographic and inflammatory parameters are synthesized in Table 3, Table 4, Table 5 and Table 6, respectively, for gaseous ozone, ozonate water, ozonate oil and ozone gel.

Due to the variability of the studies, the limited data on periodontal parameters, and the various types of ozone used, it was not possible to analyze the data at a statistical level and perform a meta-analysis.

### 3.4. Quality Assessment of the Included Studies

The risk of bias in the non-randomized clinical trials was assessed using the ROBINS-I (“Risk Of Bias In Non-randomized Studies-of Interventions”) tool [28].

The quality assessment of the included studies are summarized in Table 7

An important risk related to confounding bias is smoking status and smoking history, as reported by several studies [57,58].

In particular, McKenna, Ranjith, Kshitish, Yilmaz, Nicolini, Nardi and Ghandi [42,44,45,47,48,55,56] excluded smokers of any kind, specifying this in their exclusion criteria; in contrast, other authors such as Rapone et al. excluded subjects who smoked more than 10 cigarettes per day [40]. Isler included smokers, but it is not clear whether their study only included those who smoke fewer than 10 cigarettes per day [52].

Additionally, some authors such as Al Abashneh, Hayakumo and Tasdemir did not specify anything about smoking generating a significant bias in their results [43,46,49].

Nardi also included the inclusion criterion of participants who have a plaque index (PI) ≥ 35% and Gingival index (GI) ≥ 35%, which could be another confounding factor [48].

## 4. Discussion

The gold standard for periodontal treatment of periodontitis is scaling and root planing (SRP); however, many additional remedies have been described including the use of ozone [21,22,23,59,60].

Ozone is used in various branches of medicine, in particular in dentistry, due to its antimicrobial properties as it has been observed to have an effect on bacteria, viruses and fungi [3,61].

Regarding its antibacterial properties, ozone has been used in various formulations from gas to irrigation with ozonized water to the application of ozonized oil and ozone-based gel for the treatment of periodontal diseases and peri-implantitis according to many authors [40,41,42,43,44,45,46,47,48,49,50,51,52,53,54,55,56].

### 4.1. Clinical Periodontal and Peri-Implant Parameters in Patients Treated with Gaseous Ozone

The topical administration of ozone in gaseous form can be performed with an open system or with an aspiration system to prevent patients from inhaling the gas and therefore causing adverse effects [3].

Dengizek and Tasdemir did not find significant differences in CAL values between the groups treated without ozone and the group treated with gaseous ozone [41,43]. However, they considered short follow-up periods of 1 month and 3 months, respectively. In contrast, Rapone and Isler found statistically significant differences among the groups, and Isler considered a follow-up of 12 months [40,52].

Several results were discovered regarding BOP and PPD. In particular, Tasdemir did not find significant differences between SRP alone and SRP combined with ozone therapy at 3 months follow up [43], whereas Rapone described better results for SRP combined with ozone therapy in periodontitis compared to SRP alone at follow-up after 3 months [40]. However, the differences obtained could be caused by the different type of ozone application used. Tasdemir [43] considered the application of topical ozone in periodontal pockets twice a week for 2 weeks during active periodontal therapy. In contrast, Rapone proposed a protocol that envisaged both the use of ozonated water and cycles of gaseous ozone in pathological pockets [40].

McKenna instead compared treatment with O_3_ + NaCl with treatment with O_3_ + H_2_O_2_ and found that they were equally the most effective treatments, whereas O_2_ + NaCl was the least effective in controlling the bleeding around implants [55].

Yilmaz compared three different groups: an SRP only group, an SRP + ozone group and an SRP + laser group, and only the laser + SRP group showed statistically significant differences among the groups [44].

Tasdemir and Dengizek found no statistically significant differences regarding PI among the groups. McKenna found that treatment with O_3_ + NaCl and O_3_ + H_2_O_2_ were equally the most effective treatments for GI and PI, whereas O_2_ + NaCl was the least effective [41,43].

### 4.2. Clinical Periodontal and Peri-Implant Parameters in Patients Treated with Ozonated Water

Ozonated water was a very effective oral disinfectant thanks to its marked action against bacteria, fungi and viruses. Furthermore, it is cheaper than other oral chemical products. The aqueous form of ozone has proved to be less effective than the gaseous form but is more applicable both for addressing minor side effects (indeed, ozone gas has toxic effects if inhaled) and for its ease of use [3].

Ranjith, in a triple-blind RCT, compared CAL, PPD and IL-1b values in a group treated with SRP + ozonized water irrigation with a group treated with SRP + saline irrigation and showed that SRP + ozonized water irrigation possessed better results compared to SRP+ saline irrigation after a follow-up period of 4 weeks [42].

Al Abashneh [49] showed that treatment with ozonated water irrigation and SRP compared to treatment with SRP with saline irrigation showed no statistically significant differences regarding GI after a follow-up period of 3 months, and the same results were described by Nicolini regarding PI [47].

Different results are probably obtained due to the fact that each author used different protocols to compare different groups.

For example, Kshitish considered one group treated with ozonated water and one with chlorhexidine gluconate for a period of 18 days divided into two time intervals with a 4-day washout period followed by a second 7-day time interval. He showed a BOP, PI and GI reduction using ozonated water and thus concluded that ozone could be a valid alternative for management of periodontitis [45]. Furthermore, Vasthavi showed statistically significant differences between groups regarding PI and GI [54].

### 4.3. Clinical Periodontal and Peri-Implant Parameters in Patients Treated with Ozonated Olive or Sunflower Oil

Ozonated sunflower or olive oil has been proven to be an excellent antimicrobial agent and has been proven to be particularly effective against Staphylococci, Streptococci, Enterococci, Pseudomonas, Escherichia coli and, above all, against Mycobacteria [61,62,63,64].

This type of treatment can be used after in-office treatment with ozone gas or ozonated water or even simply at home using a 25 gauge blunt needle [3].

The use of ozonated olive oil in the treatment of periodontitis has been evaluated by many studies.

Some authors have shown that the addition of ozonated olive oil with SRP has proven benefits, while others have not.

Notably, Patel and Nardi described that ozonated olive oil and SRP improve BOP and also PPD [48,50], whereas Nambiar found no difference in BOP and PPD between treatments using SRP with olive oil and chlorhexidine groups with treatment using SRP [53].

Nardi evaluated salivary MMP-8 metalloproteinase levels in the periodontium, demonstrating that adding ozonated olive oil mouthwash to non-surgical periodontal treatment produced better results on salivary MMP-8 reduction compared to non-surgical treatment alone [50].

In some studies, Gandhi [56] and Nambiar [53], found no significant difference in the addition of ozonated olive oil to SRP compared to the use of chlorhexidine for improved PI, GI, PD, CAL and reduced periodontopathogenic bacteria.

However, other authors such as Patel have described that the addition of ozonated olive oil to SRP led to significant improvements in CAL, GI and PI [48].

### 4.4. Clinical Periodontal and Peri-Implant Parameters in Patients Treated with Ozonated Gel

Ozonated gel is another available formulation of ozone, but it has only been analyzed by Colombo.

Colombo evaluated in an RCT the difference between ozonated gel + SRP, compared to SRP + chlorhexidine gel with follow-up periods of 1 month and 3 months regarding PPD, CAL, GI, PI and BoP values; the results showed that the use of the ozonated gel in addition to SRP had no greater efficacy than treatment involving SRP + chlorhexidine [51].

Better results were obtained with SRP + chlorhexidine regarding CAL and GI at follow up after 3 months [51].

## 5. Conclusions

The studies included in this systematic review show different results regarding the influence of ozone related to the improving periodontal treatment with or without SRP.

Furthermore, the heterogeneity of the included studies and their risk of bias should be considered. Ozone shows encouraging therapeutic effects with regard to periodontal and peri-implant disease. However, the gold standard for the treatment of periodontitis remains SRP, whereas studies with sufficiently large samples and with low risks of bias are necessary to aid in our understanding of the effectiveness of ozone in the different formulations proposed regarding periodontal parameters.

Another important factor to consider is that, among the various formulations in which ozone is available, it should be borne in mind that, although the gaseous formulation may seem to be the most effective, it is also the most dangerous as inhaling ozone can be toxic for the respiratory system and other organs.

The complications caused by ozone therapy are rare but possible and range from minor side effects such as rhinitis, cough, headache, nausea and vomiting to more serious effects such as circulation and respiratory problems, heart problems and even ischemia.

## Figures and Tables

**Table 1:** Studies excluded along with reasons for exclusion.

Authors, Year	Reason for Exclusion
Rapone, 2020 [29]	Did not meet inclusion criteria
Suh, 2019 [7]	Did not meet inclusion criteria
Elvis, 2011 [30]	Did not meet inclusion criteria
Bocci, 2009 [31]	Did not meet inclusion criteria
Abreu-Villela, 2021 [32]	Was not a human study
Lio, 2020 [33]	Did not meet inclusion criteria
Deepthi,2020 [34]	Did not meet inclusion criteria
Srikanth, 2013 [35]	Did not meet inclusion criteria
Butera, 2023 [36]	Did not meet inclusion criteria
Tonon, 2021 [37]	Did not meet inclusion criteria
Piva, 2020 [38]	Contained no periodontal parameters
Moreo, 2020 [39]	Contained no periodontal parameters

**Table 2:** Characteristics of the studies included in the present systematic review: author(s) and year of publication; study design; population, sample size, mean age; type of ozone used; Main results and Conclusions. Periodontal and peri-implant clinical, radiographic and crevicular parameters, including clinical attachment loss (CAL), probing depth (PPD), bleeding on probing (BoP), plaque index (PI), gingival index (GI), marginal bone levels (MBL), cytokine profile including interlukine-1b (IL-1b); matrix metalloproteinase (MMP-8) and periodontal treatment with scaling and Root Planing (SRP); Full-Mouth mechanical debridement (FMMD) and Test Group (TG); Control Group (CG); Gaseous Ozone Therapy (GOT); Right Quadrants (RQ); Left Quadrants (LQ); mechanical debridement (MT).

Authors, Year of Reference Study and Design	Population, Sample Size and Mean Age	Type of Ozone Used	Time Points	Main Results
Rapone, 2022 [40] RCT	45 TG (SRP + GOT) 45 CG (SRP) 51.56 ± 10.35	Gaseous ozone	3 months 6 months	Statistically significant difference between TG and CG in CAL (*p* ≤ 0.0001), PPD (*p* ≤ 0.0001) and BoP (*p* ≤ 0.0001) scores.
Dengizek, 2019 [41] RCT	20 TG: SRP + 3 watts gaseous ozone 20 CG: SRP + placebo 42.4 ± 6.7	Gaseous ozone	Baseline 1 month	PI, GI, PD and CAL scores were similar for both groups (*p* > 0.05).
McKenna, 2013 [25] RCT double blind	80 implants divided in 4 groups G1: O_2_ + saline (NaCl 0.9%) G2: O_2_ + H_2_O_2_ (3%) G3: O_3_ + saline G4: O_3_ + H_2_O_2_ 60 ± 7.7	Gaseous ozone	Baseline 7th day 14th day 21st day	Significant differences were found among the treatments (*p* < 0.01) for PI (F = 16.68), GI (F = 7.86) and BoP (F = 18.42) (G3 and G4 with respect to G1 and G2).
Ranjith, 2022 [42] RCT triple blind	25 TG: SRP + ozonized water irrigation 25 CG: SRP + saline irrigation	Ozonized water	Baseline 4 weeks	Ozone water irrigation resulted in significant improvement in all clinical parameters except PPD. Salivary interleukin 1 beta also reduced significantly in the test group after therapy.
Tasdemir, 2019 [43] RCT	18 TG: SRP + Topical GOT 18 CG: SRP + Topical GOT without starting Ozone generator 43.7	Gaseous ozone	Baseline 3 months	PI, GI, PD, BoP and CAL were similar for both groups. All inflammatory parameters (PTX-3, Hs-CRP and IL-1) were reduced at 3-month follow-up. Only the decrease in PTX-3 levels between baseline and 3-month follow-up was statistically significant.
Yilmaz, 2013 [44] RCT	10 G1: SRP + ER (YAG laser) 10 G2: SRP + topical GOT 10 G3: SRP 43 ± 5.01 (G1) 41.4 ± 8.86 (G2) 41.4 ± 4.62 (G3)	Gaseous ozone	Baseline 90th day	All treatments reduced the number of total bacteria and the proportion of obligately anaerobic microorganisms. Clinical findings, including attachment gain and PD reduction, were found to be statistically significant in favor of the SRP + Er:YAG laser group.
Kshitish, 2010 [45] RCT double blind	16 G1: SRP + Ozone irrigation 16 G2: SRP + 0.2% Chlorhexidine irrigation 20–60 years (range)	Ozonated water	Baseline 7th day 18th day	A higher percentage of plaque index (12%), gingival index (29%) and bleeding index (26%) reduction was observed using ozone irrigation compared to chlorhexidine.
Hayakumo, 2013 [46] RCT	11 TG: FMMD + NBW3 10 CG: FMMD + Tap Water 45.9 ± 14.8	Ozone nano-bubble water(NBW3), tap water (WATER)	Baseline 4 weeks 8 weeks	Reduction in PPD and the clinical attachment gain after 4 and 8 weeks in the TG were significantly greater than those in the CG. TG showed statistically significant reductions in the mean total number of bacteria in subgingival plaque during the study period.
Nicolini, 2021 [47] RCT double blind	21 TG: mouthwash of ozonated water 21 CG: mouthwash of bidistilled water 23.43 ± 3.63	Ozonated water	Baseline 24h 48h 72h 96h	Plaque Free Zone Index showed no statistical difference between Test and Control groups.
Nardi, 2020 [48] RCT	48 TG: SRP + mouthwashes based on ozonated olive oil 48 CG: SRP 30–60 years (range)	Ozonated Olive Oil	Baseline 14th day 1 month 6 months	A significant improvement in PI, BoP, PPD and salivary MMP-8 levels was observed in both groups. Efficacy of ozonated olive oil in decreasing MMP-8 level. Simultaneously, it slowed the decrease in the BoP index.
Al Habashneh, 2015 [49] RCT	20 TG: SRP + ozonated water irrigation 21 CG: SRP + distilled water irrigation 39.7 ± 13.7 (TG) 39 ± 10.2 (CG)	Ozonated water	Baseline 3 months	Statistically significant improvement in the study parameters in both groups between T0 and T1 except for gingival index.
Patel, 2012 [50] RCT double blind	20 Group A: SRP 20 Group B: SPR + topical ozonated olive oil 20 Group C: topical ozonated olive oil 20 Group D: topical chlorhexidine	Ozonated Olive Oil	Baseline 4 weeks 8 weeks	Group B resulted in a significant improvement (*p* < 0.001) in clinical parameters as well as microbiological parameters over the time frame and in comparison to the control groups. Group C showed a significant improvement (*p* < 0.001) in clinical parameters as well as microbiological parameters. There was a significant increase (*p* < 0.05) in dentinal hypersensitivity following OZO as an adjunct to scaling and root planing therapy.
Gandhi, 2019 [36] RCT double blind	25 TG (2 Quadrants): SRP + ozonated olive oil 25 CG (2 Quadrants): SRP + chlorhexidine 30–60 years (range)	Ozonated Olive Oil	Baseline 3 months	Regarding intergroup comparison, no statistically significant differences were found between the CHX and ozonated olive oil groups regarding any of the clinical and microbiological parameters at the follow-up visit.
Colombo, 2021 [51] Prospective single-group and single-center RCT	10 TG: SRP + ozone gel 10 CG: SRP + chlorhexidine gel 50.0	Ozone gel	Baseline 1 month 3 months	The use of the ozonized gel in addition to SRP did not show significant differences compared to conventional SRP + chlorhexidine.
Isler, 2018 [52] RCT	20 TG: MD + sterile saline + GOT 21 CG: MD + sterile saline 53.55 ± 8.98	Gaseous ozone	Baseline 1 month 3 months 6 months 12 months	Treatment success was obtained in 50% of the implants in the ozone group and 36.6% of the implants in the control group. However, a statistically significant difference was not observed between the groups regarding treatment success.
Nambiar, 2022 [53] RCT	30 TG: SRP + ozonated olive oil 30 CG: SRP + chlorhexidine gel 38.2	Ozonated Olive Oil	Baseline 3 months	Three months’ posttreatment, all the parameters showed significant improvement in both groups. However, the intergroup comparison failed to be statistically significant.
Vasthavi, 2020 [54] RCT	12 TG: SRP + ozonated water irrigation 12 CG: SRP + distilled water irrigation 30–65 years (range)	Ozonated water	Baseline 14th day 21st day 2 months	Significant improvement in both clinical and microbiological parameters suggests that subgingival ozonated water irrigation could be an efficient adjunct to SRP.

**Table 3:** Characteristics of the periodontal clinical, radiographic and inflammatory parameters for gaseous ozone, including clinical attachment loss (CAL), probing depth (PPD), bleeding on probing (BoP), plaque index (PI), gingival index (GI), marginal bone levels (MBL) and periodontal treatment with Scaling and Root Planing (SRP).

Periodontal Clinical and Radiographic Parameters	Author, Year of Reference Study and Design	Main Results	Considerations
CAL	Dengizek, 2019 [41] RCT	The levels of CAL were similar for both groups (*p* > 0.05).	No statistically significant difference between the groups.
CAL	Tasdemir, 2019 [43] RCT	The levels of CAL were similar for both groups.	No significant differences between the two groups at baseline or 3 months (*p* > 0.05).
CAL CAL	Rapone, 2022 [40] RCT Isler, 2018 [52] RCT	At 3 months, a statistically significant difference between groups was observed from baseline in the CAL (*p* ≤ 0.0001). At 12 months postoperatively, significant differences were observed in the CAL values for both groups compared to baseline (*p* < 0.001).	There were significant CAL differences among the groups. There were no statistically significant differences in the CAL values between the groups.
BOP	McKenna, 2013 [36] RCT double blind	Significant differences were seen among the treatments (*p* < 0.01) regarding bleeding.	O_3_ + NaCl and O_3_ + H_2_O_2_ were equally the most effective treatments, whereas O_2_ + NaCl was the least effective in controlling the bleeding around implants.
BOP	Rapone, 2022 [40] RCT	At 3 months, a statistically significant difference in the BOP (*p* ≤ 0.0001) was observed between the groups.	SRP + ozone therapy improved BOP compared with SRP alone.
BOP	Tasdemir, 2019 [43] RCT	Significant decreases in percentage of BOP were determined in both groups after periodontal treatment.	No significant differences between the two groups at baseline or 3 months (*p* > 0.05).
PPD	Tasdemir, 2019 [43] RCT	Significant decreases in PD were determined in both groups after periodontal treatment.	No significant differences between the two groups at baseline or 3 months (*p* > 0.05).
PPD	Yilmaz, 2013 [44] RCT	Statistically significant differences were detected in favor of Group 1 in the double comparisons of Group 1–Group 2, Group 1–Group 3 and Group 2–Group 3 (*p* = 0.002; *p* = 0.09; and *p* = 0.365, respectively).	SRP + Er:YAG laser was more effective in PPD reduction compared to SRP + ozone or SRP only.
PPD	Rapone, 2022 [40] RCT	At 3 months, a statistically significant difference in the PPD (*p* ≤ 0.0001) was observed between the groups.	SRP + ozone therapy improved PPD compared to SRP only.
PI	Dengizek, 2019 [41] RCT	PI score was similar for both groups (*p* > 0.05).	No statistically significant difference between both groups.
PI	McKenna, 2013 [55] RCT double blind	Significant differences were seen among the treatments (*p* < 0.01) for plaque (F = 16.68).	In order of increasing effectiveness, we observed O_2_ + NaCl, O_2_ + H_2_O_2_, O_3_ + NaCl, and O_3_ + H_2_O_2_. O3 with or without H_2_O_2_ can reduce the development of peri-implant mucositis.
PI	Tasdemir, 2019 [43] RCT	Similar decreases in PI indicators of oral hygiene were observed on both sides.	This finding is not surprising since oral hygiene motivations were repeated after all sessions.
GI	Dengizek, 2019 [41] RCT	GI score was similar for both groups (*p* > 0.05).	No statistically significant difference between both groups.
GI	McKenna, 2013 [55] RCT double blind	Significant differences were seen among the treatments (*p* < 0.01) regarding gingival index (F = 7.86).	In order of increasing effectiveness, we observed O_2_ + NaCl, O_2_ + H_2_O_2_, O_3_ + NaCl, and O_3_ + H_2_O_2_. O_3_ with or without H_2_O_2_ can reduce the development Of peri-implant mucositis.
PD	Isler, 2018 [52] RCT	A significant difference was only detected in the PD values between the groups at the 3-month follow- up period in favor of the ozone group (*p* < 0.05).	At 12 months postoperatively, significant differences were observed in the PD values for both groups compared to baseline (*p* < 0.001).

**Table 4:** Characteristics of the periodontal clinical, radiographic and inflammatory parameters for ozonate water, including clinical attachment loss (CAL), probing depth (PPD), bleeding on probing (BoP), plaque index (PI), gingival index (GI), marginal bone levels (MBL), Interlukine-1b (IL-1b) and periodontal treatment with Scaling and Root Planing (SRP).

Periodontal Clinical and Radiographic Parameters	Author, Year of Reference Study and Design	Main Results	Considerations
CAL	Ranjith, 2022 [42] RCT triple blind	The mean clinical attachment gain in moderate and deep pockets was significantly greater in the test group (*p* < 0.01).	Significant clinical attachment gain may be indicative of better tissue regeneration with the use of ozone therapy compared to saline.
BOP	Kshitish, 2010 [45] RCT double blind	A higher percentage of bleeding index (26%) reduction was observed using ozone irrigation compared to chlorhexidine.	Ozone may be considered an alternative management strategy due to its powerful ability to inactivate microorganisms.
PPD	Ranjith, 2022 [42] RCT triple blind	Ozone water irrigation resulted in significant reduction in pocket depth in deep pockets (*p* = 0.01) and the number of sites with a pocket depth ≥ 4 mm with bleeding on probing.	PPD reduction was statistically significant for deep pockets (>7 mm) and for pockets with depth ≥ 4 mm with bleeding on probing.
PPD	Hayakumo, 2013 [46] RCT	There were statistically significant improvements in the full-mouth mean PPD in both groups from baseline to 4 weeks and 8 weeks. The differences in the full-mouth mean PPD were not statistically significant at each follow-up visit.	The reductions in mean PPD from baseline to 4 and 8 weeks in the NBW3 group were significantly greater than those in the water group.
PI	Kshitish, 2010 [45] RCT double blind	A higher percentage of plaque index (12%) reduction was observed using ozone irrigation compared to chlorhexidine.	Ozone may be considered an alternative management strategy due to its powerful ability to inactivate microorganisms.
PI	Nicolini, 2021 [47] RCT double blind	Both groups presented a very similar pattern of plaque formation according to the Plaque Free Zone Index at all time points.	No statistically significant differences among the groups.
GI	Al Habashneh, 2015 [49] RCT	The decrease sin gingival indexes were comparable in the two treatment groups.	Ozonated water irrigation as an adjunctive therapy to SRP produced no statistically significant benefit compared to SRP with distilled water irrigation.
GI	Kshitish, 2010 [45] RCT double blind	A higher percentage of gingival index (29%) reduction was observed using ozone irrigation compared to chlorhexidine.	Ozone may be considered an alternative management strategy due to its powerful ability to inactivate microorganisms.
IL-1b	Ranjith, 2022 [42] RCT triple blind	Reduction in salivary IL1 β was noticed 1 month after ozone water irrigation.	Salivary interleukin 1 beta reduced significantly in the test group after therapy.
PI	Vasthavi, 2020 [54] RCT	The mean PI scores of TG at the baseline and after 2 months were 2.505 ± 0.318 and 1.416 ± 0.372, respectively, showing a statistically significant difference (*p* < 0.001).	The mean difference in scores between the groups was not statistically significant.
GI	Vasthavi, 2020 [54] RCT	The mean GI scores of TG at the baseline and after 2 months were 2.569 ± 0.336 and 1.512 ± 0.406, respectively, showing a statistically significant difference (*p* < 0.001).	The mean difference in scores between the groups was statistically significant.
PPD	Vasthavi, 2020 [54] RCT	The mean PPD scores of TG at the baseline and after 2 months were 6.833 ± 1.193 and 4.500 ± 0.797, respectively, showing a statistically significant difference (*p* < 0.001).	The mean difference in scores between the groups was statistically significant.

**Table 5:** Characteristics of periodontal clinical, radiographic parameters and inflammatory parameters for ozonate oil, including clinical attachment loss (CAL), probing depth (PPD), bleeding on probing (BoP), plaque index (PI), gingival index (GI), marginal bone levels (MBL), matrix metalloproteinase (MMP-8) and periodontal treatment with Scaling and Root Planing (SRP).

Periodontal Clinical and Radiographic Parameters	Author, Year of Reference Study and Design	Main Results	Considerations
BOP BOP BOP	Patel, 2012 [50] RCT double blind Nardi, 2020 [48] RCT Nambiar, 2022 [53] RCT	The adjunctive use of the OZO with SRP resulted in a significant improvement (*p* < 0.001) in BOP. A significant improvement in BoP was observed in both groups. There was a non-statistically significant reduction in both groups, but not between the two groups.	Ozonate olive oil + SRP improved BOP. The ozonated olive oil mouthwash slowed the decrease in BoP index at the beginning of treatment. No differences between ozone olive oil + SRP and clorexidine group + SRP.
PPD PPD PPD PI	Nardi, 2020 [48] RCT Patel, 2012 [50] RCT double blind Nambiar, 2022 [53] RCT Patel, 2012 [50] RCT double blind	Groups showed significantly different variances regarding PPD (*p* < 0.001 in Levene’s test). The adjunctive use of the OZO with SRP resulted in a significant improvement (*p* < 0.001) in PPD. There was a non-statistically significant reduction in both groups, but not between the two groups. The adjunctive use of the OZO with SRP resulted in a significant improvement (*p* < 0.001) in PI.	PPD decreased both in the study and control group with no statistical significance. Ozonate olive oil + SRP improved PPD. No differences between ozone olive oil + SRP and clorexidine group + SRP. Ozonate olive oil + SRP improved PI.
PI	Nardi, 2020 [48] RCT	A significant improvement in PI was observed in both groups.	Ozonated olive oil slowed the decrease in PI index at the beginning of treatment (at T1).
MMP-8	Nardi, 2020 [48] RCT	This study showed the efficacy of ozonated olive oil in decreasing MMP-8 levels.	SRP + ozonated oil led to a significant and faster reduction in saliva MMP-8 concentrations in patients with periodontitis.
PPD GI	Gandhi, 2019 [56] RCT double blind Patel, 2012 [50] RCT double blind	No significant difference was found in the efficacy of ozonated olive oil and CHX in improving the PD. The adjunctive use of the OZO with SRP resulted in a significant improvement (*p* < 0.001) in GI.	No statistically significant differences were found between the CHX and ozonated olive oil groups regarding any of the clinical parameters at the follow-up visit. Ozonate olive oil + SRP improved GI.
CAL CAL CAL	Gandhi, 2019 [56] RCT double blind Nambiar, 2022 [53] RCT Patel, 2012 [50] RCT double blind	No significant difference was found in the efficacy of ozonated olive oil and CHX in improving clinical attachment levels. There was a non-statistically significant reduction in both groups, but not between the two groups. The adjunctive use of the OZO with SRP resulted in a significant improvement (*p* < 0.001) in CAL.	No statistically significant differences were found between the CHX and ozonated olive oil groups regarding any of the clinical and microbiological parameters at the follow-up visit. No differences between ozone olive oil + SRP and clorexidine group + SRP. Ozonate olive oil + SRP improved CAL.

**Table 6:** Characteristics of periodontal clinical, radiographic parameters and inflammatory parameters for ozone gel, including clinical attachment loss (CAL), probing depth (PPD), bleeding on probing (BoP) and periodontal treatment with Scaling and Root Planing (SRP).

Periodontal Clinical and Radiographic Parameters	Author, Year of Reference and Study Design	Main Results	Considerations
CAL	Colombo, 2021 [51] Prospective single-group and single-center RCT	Significant intragroup differences were found between each time point for the sites treated with SRP plus chlorhexidine (*p* < 0.05), whereas for ozone, a significant improvement was found at T1 but not at T2.	A significant intergroup difference was found between the sites at T2 (*p* < 0.05).
BOP	Colombo, 2021 [51] Prospective single-group and single-center RCT	Significant intragroup differences were found between each time point for both groups.	Significant intergroup differences were found between the sites (*p* > 0.05).
PPD	Colombo, 2021 [51] Prospective single-group and single-center RCT	Significant intragroup differences were found between each time point for both groups.	Significant intergroup differences were found between the sites (*p* > 0.05).

**Table 7:** Risk of bias in the studies included in the systematic review. The response options regarding biases included Yes (Y), Probably yes (PY), Probably no (PN), No (N) and No information (NI). “Y” indicated a low risk of bias, “PY” indicated a moderate risk of bias, “PN” indicated a serious risk of bias, “N” indicated a critical risk of bias and NI indicated that was no information related to bias.

Studies	Bias due to Confounding	Bias in Selection of Participants	Bias in Measurement Classification of Interventions	Bias due to Deviations from Intended Interventions	Bias due to Missing Data	Bias in Measurement of Outcomes	Bias due to Selection of the Reported Result
Rapone, 2022 [40]	Y/**PY**/ PN/N	**Y**/PY/ PN/N/NI	**Y**/PY/ PN/N/NI	**Y**/PY/ PN/N/NI	**Y**/PY/ PN/N/NI	**Y**/PY/ PN/N/NI	**Y**/PY/ PN/N/NI
Dengizek, 2019 [41]	Y/**PY**/ PN/N	**Y**/PY/ PN/N/NI	**Y**/PY/ PN/N/NI	**Y**/PY/ PN/N/NI	**Y**/PY/ PN/N/NI	**Y**/PY/ PN/N/NI	**Y**/PY/ PN/N/NI
McKenna, 2013 [55]	Y/**PY**/ PN/N	Y/**PY**/ PN/N/NI	**Y**/PY/ PN/N/NI	**Y**/PY/ PN/N/NI	**Y**/PY/ PN/N/NI	**Y**/PY/ PN/N/NI	**Y**/PY/ PN/N/NI
Ranjith, 2022 [42]	**Y**/PY/ PN/N	Y/**PY**/ PN/N/NI	**Y**/PY/ PN/N/NI	**Y**/PY/ PN/N/NI	**Y**/PY/ PN/N/NI	**Y**/PY/ PN/N/NI	**Y**/PY/ PN/N/NI
Nambiar, 2022 [53]	**Y**/PY/ PN/N	Y/**PY**/ PN/N/NI	**Y**/PY/ PN/N/NI	**Y**/PY/ PN/N/NI	**Y**/PY/ PN/N/NI	**Y**/PY/ PN/N/NI	**Y**/PY/ PN/N/NI
Vasthavi, 2020 [54]	**Y**/PY/ PN/N	Y/**PY**/ PN/N/NI	**Y**/PY/ PN/N/NI	**Y**/PY/ PN/N/NI	**Y**/PY/ PN/N/NI	**Y**/PY/ PN/N/NI	**Y**/PY/ PN/N/NI
Yilmaz, 2013 [43]	**Y**/PY/ PN/N	Y/PY/ **PN**/N/NI	**Y**/PY/ PN/N/NI	**Y**/PY/ PN/N/NI	**Y**/PY/ PN/N/NI	Y/**PY**/ PN/N/NI	**Y**/PY/ PN/N/NI
Yilmaz, 2013 [44]	**Y**/PY/ PN/N	Y/**PY**/ PN/N/NI	**Y**/PY/ PN/N/NI	**Y**/PY/ PN/N/NI	**Y**/PY/ PN/N/NI	**Y**/PY/ PN/N/NI	**Y**/PY/ PN/N/NI
Kshitish, 2010 [45]	**Y**/PY/ PN/N	Y/**PY**/ PN/N/NI	**Y**/PY/ PN/N/NI	**Y**/PY/ PN/N/NI	**Y**/PY/ PN/N/NI	**Y**/PY/ PN/N/NI	**Y**/PY/ PN/N/NI
Hayakumo, 2013 [46]	**Y**/PY/ PN/N	Y/**PY**/ PN/N/NI	**Y**/PY/ PN/N/NI	**Y**/PY/ PN/N/NI	**Y**/PY/ PN/N/NI	Y/**PY**/ PN/N/NI	**Y**/PY/ PN/N/NI
Nicolini, 2021 [47]	**Y**/PY/ PN/N	Y/**PY**/ PN/N/NI	**Y**/PY/ PN/N/NI	**Y**/PY/ PN/N/NI	**Y**/PY/ PN/N/NI	**Y**/PY/ PN/N/NI	**Y**/PY/ PN/N/NI
Nardi, 2020 [48]	Y/**PY**/ PN/N	Y/**PY**/ PN/N/NI	**Y**/PY/ PN/N/NI	**Y**/PY/ PN/N/NI	**Y**/PY/ PN/N/NI	**Y**/PY/ PN/N/NI	**Y**/PY/ PN/N/NI
Al Abashneh, 2015 [49]	**Y**/PY/ PN/N	Y/**PY**/ PN/N/NI	**Y**/PY/ PN/N/NI	**Y**/PY/ PN/N/NI	**Y**/PY/ PN/N/NI	Y/**PY**/ PN/N/NI	**Y**/PY/ PN/N/NI
Patel, 2012 [50]	**Y**/PY/ PN/N	**Y**/PY/ PN/N/NI	**Y**/PY/ PN/N/NI	**Y**/PY/ PN/N/NI	**Y**/PY/ PN/N/NI	**Y**/PY/ PN/N/NI	**Y**/PY/ PN/N/NI
Gandhi, 2019 [56]	**Y**/PY/ PN/N	Y/**PY**/ PN/N/NI	**Y**/PY/ PN/N/NI	**Y**/PY/ PN/N/NI	**Y**/PY/ PN/N/NI	**Y**/PY/ PN/N/NI	**Y**/PY/ PN/N/NI
Colombo, 2021 [51]	**Y**/PY/ PN/N	Y/**PY**/ PN/N/NI	**Y**/PY/ PN/N/NI	**Y**/PY/ PN/N/NI	**Y**/PY/ PN/N/NI	**Y**/PY/ PN/N/NI	**Y**/PY/ PN/N/NI
Isler, 2018 [52]	**Y**/PY/ PN/N	Y/**PY**/ PN/N/NI	**Y**/PY/ PN/N/NI	**Y**/PY/ PN/N/NI	**Y**/PY/ PN/N/NI	**Y**/PY/ PN/N/NI	**Y**/PY/ PN/N/NI
Risk of bias judgements	**MODERATE**	**SERIOUS**	**LOW**	**LOW**	**LOW**	**MODERATE**	LOW

## Data Availability

All data generated or analyzed during this study are included in this article.
